# Unraveling Effects and Pharmacological Mechanisms of Phellodendrine on Inflammatory Bowel Disease

**DOI:** 10.3390/biom16081092

**Published:** 2026-07-26

**Authors:** Yufeng Xie, Ziyi Zhou, Xuqianzi Wu, Jiayin Teng, Xiaorun Zhang, Yue Sun, Lixin Chen, Lijian Ding, Wei Yuan

**Affiliations:** 1 Jiangxi Provincial Key Laboratory of Synthetic Pharmaceutical Chemistry, School of Geography and Environmental Engineering, Gannan Normal University, Ganzhou 341000, China; 1241144005@gnnu.edu.cn (Y.X.);; 2 School of Pharmacy, Health Science Center, Ningbo University, Ningbo 315211, China

**Keywords:** Phellodendrine, inflammatory bowel disease, anti-inflammation, intestinal barrier, gut microbiota, oxidative stress

## Abstract

Inflammatory bowel disease (IBD) is characterized by chronic inflammation of the gastrointestinal tract. Current treatments, including anti-inflammatory drugs and biologics, often have limited efficacy and significant side effects, highlighting the need for novel therapeutic approaches. Phellodendrine (PHE) is a characteristic ingredient of *Phellodendri chinensis*, yet its effects and mechanisms on IBD remain elusive. The present study evaluated the potential of PHE for preventing dextran sulfate sodium-induced IBD in zebrafish. PHE effectively reduced inflammatory cell infiltration and modulated polarized macrophages. The qPCR results further confirmed the down-regulation of pro-inflammatory genes and up-regulation of anti-inflammatory factors. Consequently, PHE promoted the resolution of IBD inflammation. PHE also restored intestinal barrier integrity by enhancing MUC2 expression, increasing goblet cell counts, and reducing intestinal permeability of both chemical and physical barriers. In addition, PHE was associated with alterations in the gut microbiome, including a reduction in potentially pathogenic microbes and an increase in beneficial microbial populations. PHE also alleviated oxidative stress. Network pharmacology suggested the potential involvement of the IL-17 signaling pathway, the lipid and atherosclerosis pathway, and the TNF signaling pathway in the preventive effects of PHE against intestinal inflammation in the zebrafish model. In vivo gene expression analysis suggested that JUN, PTGS2, IL1B, DRD2, CALM1, and HSP90AA1 may serve as putative targets of PHE. Collectively, our results indicate that PHE demonstrates potential anti-inflammatory and barrier-protective activities in a zebrafish model of intestinal inflammation. The pharmacological mechanisms by which PHE restores intestinal barriers (microbial, chemical, physical, and immune barriers) include resolving inflammation, decreasing ROS production, and enhancing lipid accumulation in the lumen overlying the intestinal mucus barrier. This study provides novel insights into the preventive effects of PHE against intestinal inflammation in a zebrafish model, suggesting its potential as a candidate for further investigation.

## 1. Introduction

Inflammatory bowel disease (IBD) is a pathology of the gastrointestinal tract characterized by chronic relapsing immune activation [1,2]. The incidence of IBD has been progressively increasing on a global scale in modern society, impacting over 6.8 million people around the world [3,4]. The pathophysiology of IBD is complex, driven by a multifaceted interplay between genetic predispositions, environmental factors, epithelial, microbial, and immune system dysregulation [3]. Crucial markers of IBD encompass inflammatory cell infiltration, disruptions in both chemical and physical barriers of the intestine, alterations in the microbiota, and oxidative stress [5]. The chemical barrier overlying the intestinal epithelium is composed of a mucus layer, primarily mucin 2 (MUC2), in which are sequestered anti-microbial elements such as defensins, cathelicidins, and lysozyme [6,7,8]. Retention of these anti-microbial elements at the surface of the epithelium protects the epithelium from microbial invasion. Consequently, MUC2 is a critical element in the formation of a protective chemical barrier overlying the intestinal epithelium. Physical barriers, such as goblet cells, play a significant role in maintaining intestinal integrity by secreting mucus and other protective substances [9]. IBD is also associated with increased intestinal permeability, which allows for the translocation of bacteria and toxins into the bloodstream, further exacerbating the inflammatory response [10]. A growing body of literature has demonstrated that the alterations in gut microbial composition among individuals are intricately linked to IBD [11,12,13]. Another hallmark of IBD is oxidative stress, which results from a discrepancy between the generation of reactive oxygen species (ROS) and the inadequate capacity to detoxify them [14]. This oxidative stress contributes to tissue damage and inflammation, perpetuating the cycle of disease progression [14]. The interplay between these factors results in an intricate pathophysiological landscape that challenges effective disease management.

Regrettably, conventional clinical treatments, including aminosalicylates, corticosteroids, and immunosuppressants, have faced challenges [15]. This is because they provide only temporary alleviation of symptoms without tackling the fundamental issues of intestinal barrier dysfunction and microbiota imbalance [15,16]. Current treatments often entail considerable adverse effects, including nausea, vomiting, abdominal discomfort, and diarrhea, resulting in unsatisfactory therapeutic outcomes [15,17]. Accordingly, there is an urgent need for the development of new drugs for treating IBD with better efficacy and safety profiles. Traditional Chinese Medicine (TCM) has been a rich source of bioactive compounds with potential therapeutic effects against various diseases, including IBD; for instance, naringin, berberine, and luteolin [18]. Phellodendrine (PHE) is a kind of isoquinoline alkaloid, which is one of the key active constituents in the cortex of *Phellodendri chinensis* [19]. Despite its anti-inflammatory properties, the specific effects and underlying mechanisms of PHE on intestinal inflammation are still largely unexplored [19,20].

Animal models are indispensable for elucidating the pathophysiological mechanisms of IBD and the development of new therapeutics. Zebrafish have emerged as a valuable tool owing to their genetic similarity to humans, transparent embryos, and the ease of genetic manipulation [21,22]. Zebrafish models allow for real-time observation of intestinal inflammation and the effects of potential therapeutic compounds in vivo [23]. Zebrafish also offer unique opportunities to study the interactions between the host and microbiota, as well as the impact of environmental factors on disease progression [24]. These features make zebrafish an attractive model for investigating the efficacy and safety of novel therapeutic agents, as well as for elucidating the molecular mechanisms underlying IBD.

The present study utilized the zebrafish IBD model to explore the therapeutic potential of PHE and elucidate its mechanisms of action in alleviating IBD. Dextran sodium sulfate (DSS), which is commonly employed in experimental protocols to provoke IBD owing to its controllability, rapidity, simplicity, and reproducibility in model construction [25], was used to induce IBD in our zebrafish model. Thereby, the IBD model was established by DSS treatment. With the aid of a fluorescent genetically modified zebrafish line, inflammatory cell infiltration and macrophage polarization following co-administration of PHE were investigated. The expression levels of pro-inflammatory genes (*ikbkb*, *NF-kB p65*, and *il1β*) and the anti-inflammatory gene *tgfβ1a* were assessed using qPCR. Subsequently, both chemical and physical barriers were evaluated by examining MUC2 expression, goblet cell counts, and intestinal permeability in response to PHE intervention. Microbial populations were compared through 16S rRNA gene metataxonomic analysis. Additionally, the restoration of oxidative stress imbalance by PHE was examined. Integrative analysis using network pharmacology and in vivo validation elucidated the pathways and targets involved in the pharmacological function of PHE. Overall, this study provides a comprehensive understanding of the corresponding molecular mechanisms of PHE in the treatment of IBD in the zebrafish model.

In this study, we employed a zebrafish model of DSS-induced intestinal inflammation to assess the preventive effects of PHE. Our investigation comprehensively assessed the impact of PHE on the restoration of four intestinal barriers (microbial, chemical, physical, and immune barriers), modulation of the gut microbiome, attenuation of oxidative stress, and recovery of lipid absorption in this model. Additionally, we combined network pharmacology with in vivo gene expression analysis to identify candidate targets for future mechanistic exploration. This multi-dimensional approach provides a comprehensive characterization of the preventive effects of PHE in the zebrafish model.

## 2. Materials and Methods

### 2.1. Chemicals

PHE was obtained from Aladdin Biochemical Technology Co., Ltd. (P414351, Shanghai, China). Dextran sulfate sodium (DSS) salt (M.W. 40,000) was purchased from Bidepharm (R019173, Shanghai, China). The two dyes, Alcian Blue 8GX and Oil Red O, were sourced from Macklin (A801642, Shanghai, China) and BBI Life Sciences (A600395, Shanghai, China), respectively. Paraformaldehyde (PFA) was obtained from Sangon Biotech (A500684, Shanghai, China). Reactive oxygen species assay kits were acquired from Beyotime Biotechnology (S0033S, Shanghai, China). Low-melting agarose was purchased from Sangon Biotech (A600015, Shanghai, China).

### 2.2. Experimental Animals

The wildtype AB strain, the colorless Casper strain (roy−/−, nacre−/−), as well as Tg(*lyz*:DsRed) and Tg(*mfap4*:GFP) transgenic zebrafish were originally obtained from the China Zebrafish Resource Center (Wuhan, China). The adult zebrafish were maintained at 28 °C and fed with harvested *Artemia salina* 3 times a day in a 14 h light (8:00 AM–10:00 PM)/dark (10:00 PM–8:00 AM) cycle, with controlled temperature (28  ±  1 °C). This study was approved by the Animal Experiment Ethics Committee of Gannan Normal University (approval no. gnnu2022–0628).

### 2.3. Zebrafish IBD Modeling, Drug Treatment and Morphological Observation

DSS, a sulfated polysaccharide, is well-documented for its capacity to chemically damage the intestinal mucosa [23]. Zebrafish intestinal inflammation was induced by exposing larvae to 0.25% (*w*/*v*) DSS from 3 to 6 days post-fertilization (dpf). The DSS solution was freshly prepared each day. Each experimental group was replicated across three wells, with treatments administered continuously for 72 h and solutions refreshed every 24 h. To determine the appropriate concentration of PHE, larvae were exposed to PHE at concentrations of 1, 5, 10, 20, 40, and 80 µM concurrently with DSS treatment. Based on its efficacy in reducing neutrophil infiltration, 40 µM was selected as the optimal concentration for subsequent experiments. PHE was dissolved in dimethyl sulfoxide (DMSO), and the final DMSO concentration in all treatment groups was maintained below 0.1% (*v*/*v*). Prednisolone (PREL) served as the positive control at a final concentration of 25 mg/L [26]. Neutrophil infiltration and macrophage polarization were assessed using a fluorescent microscope (Zeiss (Oberkochen, Germany), AXIO Zoom.V16) and a confocal microscope (Leica, TCS SP8), respectively.

### 2.4. Whole Mount Alcian Blue Staining

Goblet cells are specialized epithelial cells in the intestine that produce mucus, creating a protective barrier between the host and the gut microbiota [9]. Zebrafish goblet cells could be visualized using the methodology of whole-mount Alcian blue staining [26]. At 6 dpf, zebrafish were fixed with a 4% (*w*/*v*) PFA solution overnight at 4 °C. The samples (*n* = 30) from each group were then rinsed in an acidic ethanol solution composed of 70% ethanol and 1% concentrated hydrochloric acid. The 0.1% alcian blue solution was prepared by mixing ethanol and glacial acetic acid in an 80:20 (*v*/*v*) ratio. Goblet cells were stained with alcian blue staining solution overnight at 4 °C. After the solution was discarded, the larvae were thoroughly rinsed with acidic ethanol for 3  ×  5 min to remove any residual background staining. The larvae were mounted in 1% (*w*/*v*) low-melting agarose for imaging, which was conducted using a Leica M205 FA stereomicroscope.

### 2.5. Oil Red O (Oro) Staining

The ORO dye selectively binds to intracellular lipid droplets, rendering the lipids visible as red lipid droplets under brightfield microscopy [27]. The lipid accumulation in the intestine was assessed by means of ORO staining. After fixation with 4% PFA for 12 h, the samples at 6 dpf were washed three times with phosphate-buffered saline (PBS) and subsequently equilibrated with 60% isopropanol for 5 min. An ORO stock solution was prepared in isopropanol at a concentration of 5 mg/mL, allowed to stand for 10 min at room temperature, filtered to remove any precipitates, and stored in the dark. For the staining process, this stock was diluted to 3 mg/mL with distilled water and applied for 2.5 h. To remove non-specific staining, samples were washed three times with 60% isopropanol solution for five minutes each. Final washing was achieved by rinsing with PBS. Brightfield images of lipid distribution were captured on a Leica M205 FA stereomicroscope for high-resolution visualization.

### 2.6. Intestinal Permeability Test

Intestinal permeability was assessed by measuring D-lactic acid levels in zebrafish larvae. At 6 dpf, 40 larvae from each group were collected and homogenized. Each experimental group consisted of three independent biological replicates (samples), with each replicate containing 40 pooled larvae. The content of D-lactic acid was determined using a commercial kit (A019-3-2, Nanjing Jiancheng Bioengineering Institute, Nanjing, China) according to the manufacturer’s instructions. The absorbance was read at 450 nm using a multimode plate reader (VICTOR Nivo™, PerkinElmer, Turku, Finland).

### 2.7. Biomarkers of Oxidative Stress

To evaluate oxidative stress following PHE treatment, all assays were performed after 72 h of exposure. For ROS measurement, larvae were incubated with 20 μM 2′,7′-dichlorodihydrofluorescein diacetate (DCFH-DA) at 28 °C in the dark for 60 min. After incubation, larvae were washed three times with E3 medium to remove residual probe. ROS accumulation was imaged using a Leica M205 FA stereomicroscope (Leica Microsystems, Wetzlar, Germany), and fluorescence intensity was quantified using ImageJ software (ImageJ 1.53e, bundled with Java 1.8.0_172). For biochemical assessments, 40 larvae from each group were collected after the 72 h exposure and homogenized in 0.9% (m/v) sodium chloride solution. The supernatant was isolated by centrifugation at 12,000 rpm for 10 min at 4 °C. The activities of superoxide dismutase (SOD), catalase (CAT), and glutathione peroxidase (GPx), as well as the levels of H_2_O_2_ and malondialdehyde (MDA), were measured according to the manufacturer’s instructions (Sangon Biotech, D799593 [SOD]; Nanjing Jiancheng Bioengineering Institute, A007-1-1 [CAT]; Beyotime Biotechnology, S0058 [GPx]; Sangon Biotech, D799773 [H_2_O_2_]; Shanghai Meilian Biotech (Shanghai, China), ml094962 [MDA]).

### 2.8. RNA Extraction and RT-qPCR Analysis

Total RNA from 30 zebrafish was extracted using a total RNA purification kit, according to the manufacturer’s instructions (Sangon Biotech, B518651). RNA (1 µg) was then reverse transcribed into cDNA using MightyScript Plus First Strand cDNA Synthesis Master Mix (gDNA digester) (Sangon Biotech, B639252). The amplification reactions were performed with SGExcel FastSYBR Mixture (Sangon Biotech, B532955) on a quantitative PCR apparatus, qTOWER 3G instrument (Analytik Jena, Jena, Germany). The amplification efficiency for each primer pair was determined using a standard curve from serial dilutions of cDNA, with efficiencies ranging from 90% to 110%. Melt-curve analysis was performed after each run to confirm the specificity of amplification, and a single peak was observed for each primer pair. *Eukaryotic elongation factor 1 alpha* (*ef1a*) was used as the reference gene, and its expression stability across all experimental groups was validated. Relative gene expression was calculated using the 2^−ΔΔCt^ method. All RT-qPCR primers are listed in Supplementary Table S1.

### 2.9. Microbiome Sequencing

Each group consisted of six parallel samples, each containing 10 whole zebrafish larvae at 6 dpf. The larvae were collected, washed three times with sterile PBS, and then homogenized for subsequent assays. 16S rRNA gene amplicon sequencing for the V3–V4 region was performed using primers 341F (CCTACGGGNGGCWGCAG) and 805R (GACTACHVGGGTATCTAATCC), utilizing a 2× Hieff^®^ Robust PCR Master Mix (Yeasen, Shanghai, China). The PCR products were quantified with a Qubit 4.0 DNA Assay Kit (Life Technologies, Waltham, MA, USA). Library construction employed Hieff NGS™ DNA Selection Beads (Yeasen, Shanghai, China), followed by sequencing on the Illumina MiSeq platform by Sangon Biotech (Shanghai, China). Raw sequencing data were processed as follows. Adapter sequences were removed using cutadapt. Paired-end reads were merged using PEAR based on overlap regions. Samples were demultiplexed according to barcode sequences, and read orientations were corrected. Quality filtering was performed using PRINSEQ with a 10 bp sliding window; reads were truncated when the average quality score within the window fell below 20, and sequences with ambiguous bases (N), short length, or low complexity were discarded. Operational taxonomic units (OTUs) were clustered at 97% similarity using USEARCH (version 8.0), with chimeric sequences identified and removed during the clustering process. OTU representative sequences were selected, and an OTU table was generated by mapping all quality-filtered reads to the representative sequences at 97% similarity. Taxonomic assignment was performed using the SILVA database (version 138.1) with a confidence threshold of 0.7. To account for sequencing depth differences, samples were rarefied for downstream alpha and beta diversity analyses. Data analysis and Visualization were accomplished using the cloud platform (https://ngs.sangon.com/, accessed on 25 November 2024). The raw sequences of 16S rRNA gene sequences have been archived in the NCBI Sequence Read Archive (accession number: PRJNA1198209).

### 2.10. Immunofluorescence of Frozen Intestinal Sections

Zebrafish specimens were fixed with 4% PFA overnight to preserve tissue morphology. Following fixation, the head and tail were excised to isolate the intestinal tissue, which was subsequently embedded in optimal cutting temperature (OCT) compound (Sakura Finetechnical (Tokyo, Japan), Tissue-Tek, 4583). Intestinal tissues were cut into 8 μm full-thickness sections using a cryotome (Leica, CM3050S). The sections were placed at room temperature for 30 min to allow adhesion, and then transferred to −20 °C overnight. Subsequently, the sections were fixed with PFA, then placed on a shaker, washed with 1× PBS for 10 min, followed by three washes with 0.5% PT solution, each for 10 min. To block non-specific binding, QuickBlock™ Western sealing solution (Beyotime Biotechnology, P0252) was applied at 4 °C for 30 min on a shaker. Next, the primary antibody (Anti-MUC2, Sangon Biotech, D161002), diluted 1:1000 in QuickBlock™ Western primary antibody diluent (Beyotime Biotechnology, P0023A), was added and incubated overnight at 4 °C. The next day, tissues were washed five times with 0.5% PT solution on a shaker for 20 min each time to remove unbound antibodies. The secondary antibody, Goat anti-Rabbit IgG (H + L) Highly Cross-Adsorbed Secondary Antibody, Alexa Fluor™ 647 (Invitrogen (Waltham, MA, USA), A-21245), was diluted 1:5000 and applied to the sections for overnight incubation at 4 °C, protected from light. Following this, the sections underwent five additional washes with 0.5% PT solution, again protected from light, for 20 min each. To stain the nuclei, a 10 µg/mL 4′,6-Diamidino-2-phenylindole dihydrochloride (DAPI (San Francisco, CA, USA), Roche, 10236276001) was applied at room temperature, shielded from light, for 1.5 h. Finally, the slides were washed three times with 0.5% PT solution, away from light, for 20 min each, before imaging with a Leica TCS SP8 confocal microscope using a 40× lens.

### 2.11. Potential Target Prediction and Molecular Docking Assessment

PHE-related targets were collected from PharmMapper, Swiss Target Prediction, CTD, and TCMSP databases. IBD-related targets were obtained from Genecards, TTD, and OMIM databases. Overlapping targets were identified using BioVenn and imported into the STRING database for PPI network construction. Cytoscape 3.8.2 was used for topological analysis to screen core targets. GO and KEGG enrichment analyses were performed using DAVID, with visualization of the top 5 GO terms and top 20 KEGG pathways via the bioinformatics online platform.

For molecular docking, target protein structures were retrieved from the PDB, and the PHE structure was generated using ChemDraw (version 23.1.1) and minimized with the MMFF94 force field. Docking was performed using AutoDock Vina (v1.1.2) with exhaustiveness = 8 and num_modes = 10. The binding site was defined based on co-crystallized ligand coordinates, and the docking protocol was validated by re-docking with RMSD values below 2.0 Å. Results were visualized using PyMOL 1.7.1.0 and LigPlot+ 2.2.4.

### 2.12. Statistical Analysis

Statistical analyses and graphing were performed using GraphPad Prism v8.01 (GraphPad Software, Boston, MA, USA). All results are expressed as mean ± SD. Normality of data distribution was assessed using the Shapiro–Wilk test. Homogeneity of variances was evaluated using Levene’s test. For comparisons between two groups, a two-tailed Student’s *t*-test was applied. For multiple group comparisons, one-way ANOVA was used, followed by Tukey’s post hoc test for parametric data. When heteroscedasticity was present (as determined by Levene’s test), Brown–Forsythe and Welch ANOVA tests were applied, with Tamhane’s T2 post hoc test for pairwise comparisons. For microbiome data, the Brown–Forsythe and Welch ANOVA with Tamhane’s T2 post hoc test was used to account for unequal variances. A *p*-value of less than 0.05 was considered statistically significant. Exact sample sizes (n) for each experiment are provided in the corresponding figure legends. All experiments were performed with at least three independent biological replicates.

## 3. Results

### 3.1. PHE Alleviated DSS-Induced Immune Cell Infiltration and Macrophage Polarization

**Figure 1:**
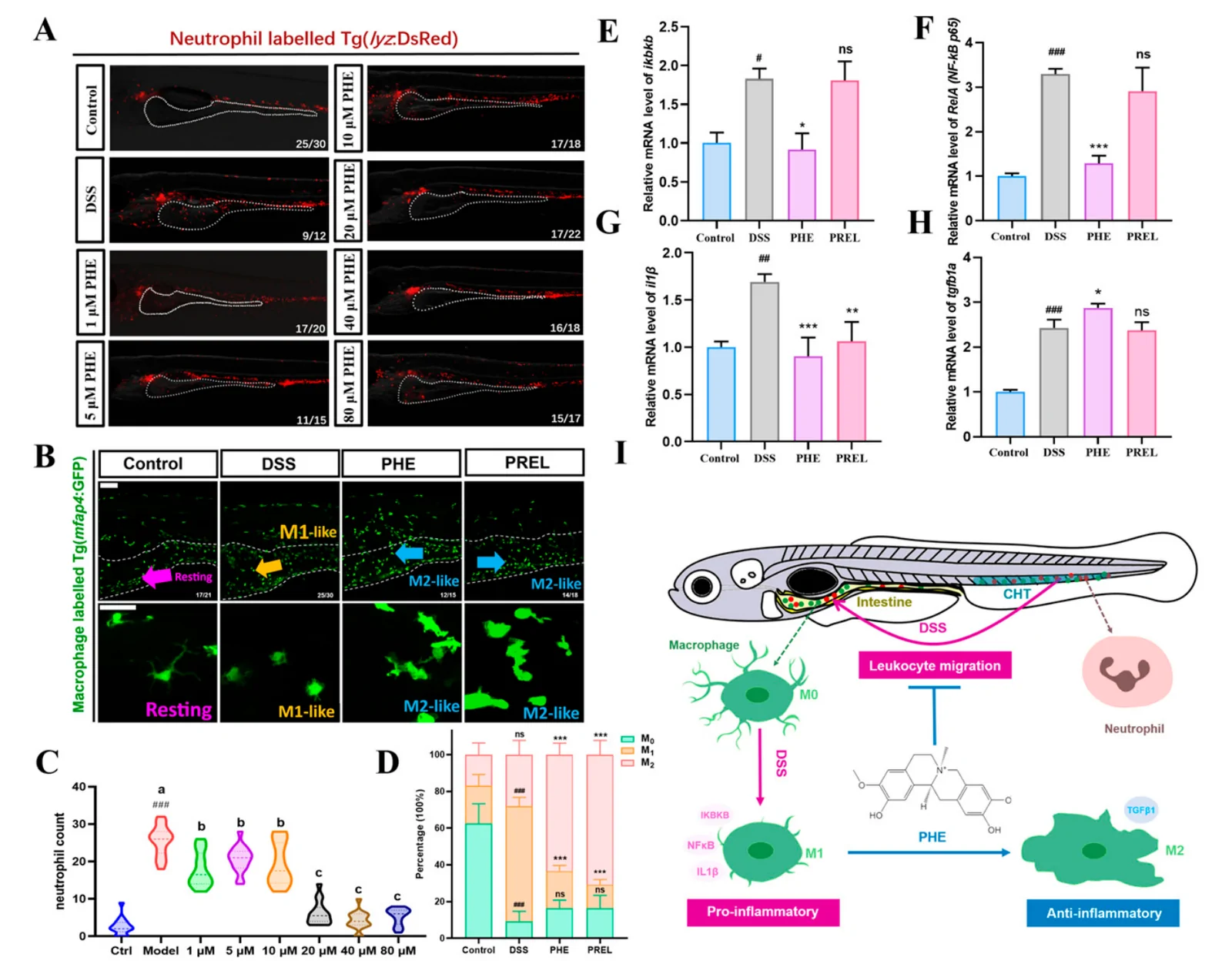
PHE reduced immune cell infiltration and altered macrophage polarization in a DSS-induced IBD model. (**A**) The optimal PHE concentration for anti-IBD effects was determined by screening various concentrations using Tg(*lyz*:DsRed) transgenic fish. (**B**) Representative confocal images showing different states of macrophages: resting state (M0, purple arrow), pro-inflammatory M1-like polarization (yellow arrow), and anti-inflammatory M2-like polarization (blue arrow) in control, DSS, PHE, and PREL groups (Scale bar = 100 μm). (**C**) Violin plots depicting the efficacy of PHE in mitigating DSS-induced inflammation at different concentrations (Significant differences between experimental groups are denoted by different letters). (**D**) Stacked bar chart illustrating the proportion of M0, M1-like, and M2-like polarized macrophages in the four groups. (**E**–**G**) Relative transcription levels of pro-inflammatory genes *ikbkb*, *NF-kB p65*, and *il1β*. (**H**) Relative transcription level of the anti-inflammatory gene tgfβ1a. (**I**) Schematic representation of the mechanism by which PHE inhibited DSS-induced inflammatory activation. Data are presented as mean ± SD (n = 3 biological replicates per group). Multiple group comparisons were performed using one-way ANOVA followed by Tukey’s post hoc test. *p*  >  0.05 (ns); * *p*  <  0.05, ** *p*  <  0.01, *** *p*  <  0.001; ### *p*  <  0.001, ## *p*  <  0.01, # *p*  <  0.05.

To investigate the preventive effects of PHE against inflammation in a zebrafish model of DSS-induced intestinal inflammation, we first determined the optimal concentration of PHE for anti-IBD effects through systematic screening of various concentrations (1, 5, 10, 20, 40, and 80 μM) using Tg(*lyz*:DsRed) transgenic fish to assess neutrophil infiltration (Figure 1A). The results indicated that 40 μM PHE yielded the most pronounced anti-IBD effects (Figure 1C), and this concentration was selected for subsequent analyses.

We also quantified the recruitment and characterized the states of monocytes in the intestine. As illustrated in Figure 1B,D, macrophages in the control group predominantly exhibited a resting state (M0), while the DSS-treated group showed a marked increase in pro-inflammatory M1-like-polarized macrophages. Notably, treatment with PHE or PREL resulted in a significant shift towards anti-inflammatory M2-like polarization, suggesting that PHE effectively modulates macrophage activation in the context of DSS-induced inflammation.

To elucidate the underlying molecular mechanisms, we analyzed the transcription levels of key pro-inflammatory and anti-inflammatory genes. The relative transcription levels of the pro-inflammatory genes ikbkb, NF-kB p65, and il1β were significantly reduced in the PHE-treated group compared to the DSS group (Figure 1E–G). Conversely, the anti-inflammatory gene *tgfb1a* exhibited a marked increase in transcription levels following PHE treatment (Figure 1H). These findings suggest that PHE not only inhibits pro-inflammatory signaling pathways but also enhances anti-inflammatory responses.

A schematic representation of the proposed mechanism by which PHE inhibits DSS-induced inflammatory activation is illustrated in Figure 1I. This model posits that PHE modulates the polarization of macrophages and consequently reduces macrophage-mediated expression of key inflammatory genes, thereby alleviating the inflammatory phenotype characteristic of IBD.

### 3.2. PHE Improved the Decline in Physical and Chemical Barriers Caused by IBD

**Figure 2:**
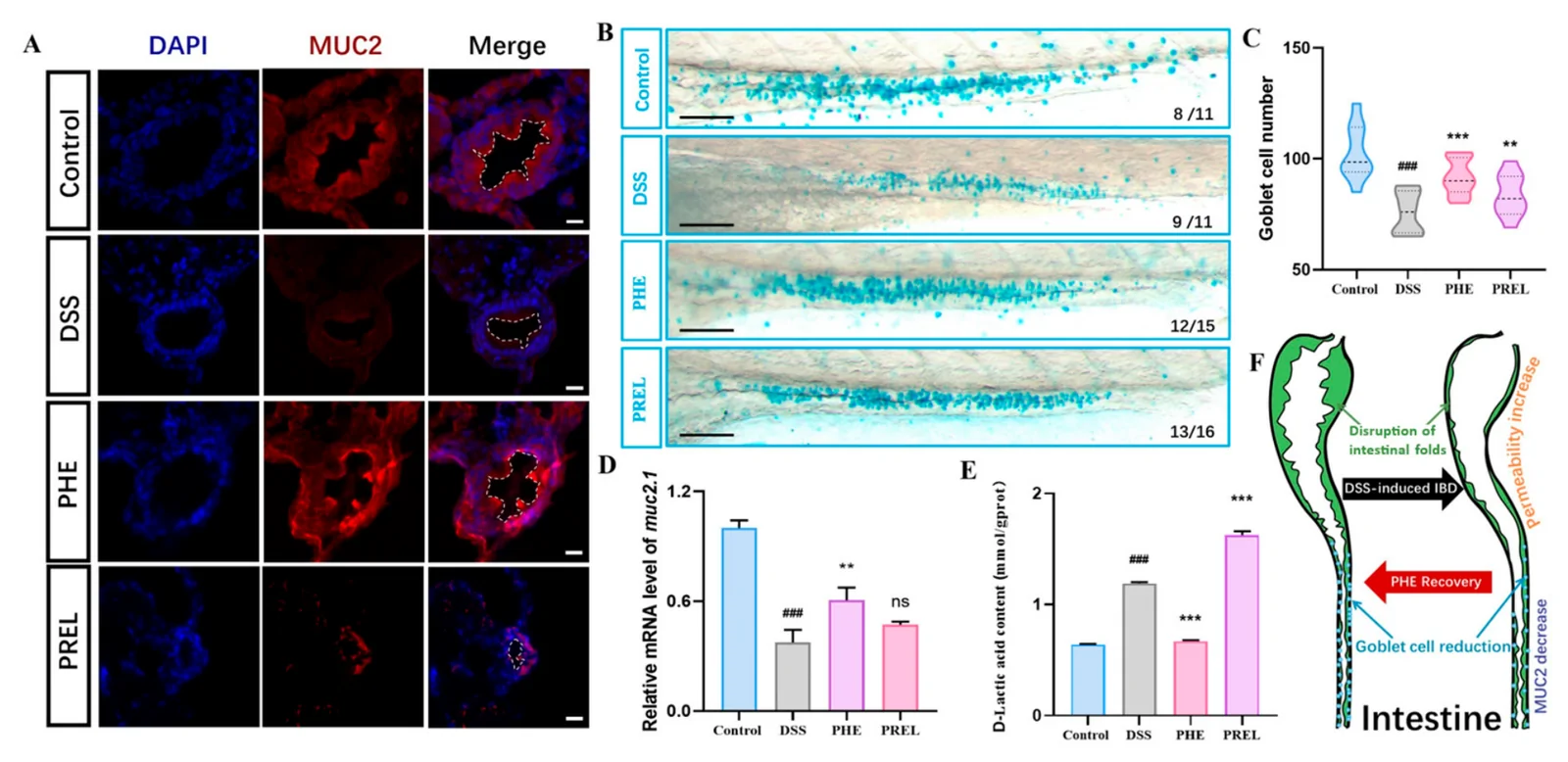
(**A**) Representative immunofluorescence staining images of MUC2 (red) and DAPI-stained nuclei (blue) in cryosections of zebrafish intestines, illustrating DSS-induced atrophy of intestinal folds. Dashed circles indicate the intestinal folds. Each immunofluorescence staining procedure was independently repeated a minimum of three times. (Scale bar = 10 μm). (**B**,**C**) Whole-mount Alcian blue staining demonstrating the protective effect of PHE against DSS-induced reduction in the physical barrier goblet cells (Scale bar = 100 μm). (**D**) qPCR analysis confirmed the regulatory change in the chemical barrier-related gene mucin *muc2.1*. (**E**) D-Lactic acid content indicating that PHE restored the DSS-induced increase in intestinal permeability. (**F**) PHE alleviation of DSS-induced intestinal fold injury, increase in permeability, reduction in goblet cells, and decrease in MUC2. Data are presented as mean ± SD (n = 3 biological replicates per group). Multiple group comparisons were performed using one-way ANOVA followed by Tukey’s post hoc test. ### *p*  <  0.001 versus the control group; *** *p*  <  0.001, ** *p*  <  0.01 versus the DSS group; ns means no significance.

**Figure 3:**
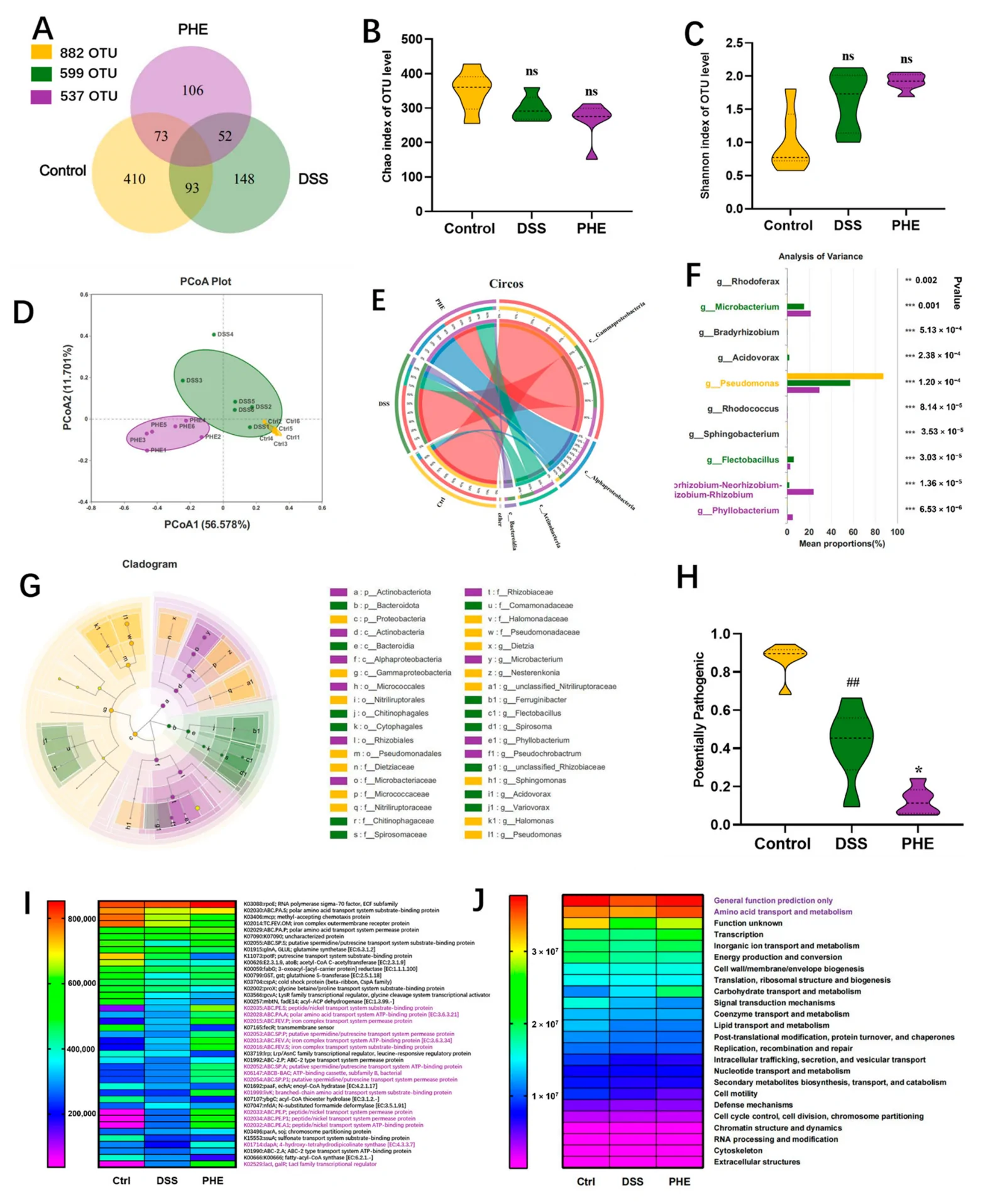
Microbiota modulation effect of PHE in DSS-induced IBD. (**A**) Venn graph among the three groups. Alpha diversity index of gut microbiota: (**B**) Chao index and (**C**) Simpson index. Beta diversity analysis: (**D**) Principal coordinate analysis (PCoA). (**E**) The circos graph showing the community analysis at the class level. (**F**) The analysis of variance at the genus level. (**G**) LEfSe analysis of microbiota. (**H**) Potentially pathogenic analysis using BugBase. (**I**,**J**) KEGG and functional annotation using PICRUSt2. Brown–Forsythe and Welch ANOVA tests. ## *p*  <  0.01 versus the control group; *** *p*  <  0.001, ** *p*  <  0.01, * *p*  <  0.05 versus the DSS group; ns means no significance.

We investigated the preventive effects of PHE against DSS-induced intestinal damage, with particular focus on the physical and chemical barriers. Immunofluorescence results indicated that DSS treatment led to significant atrophy of intestinal folds (Figure 2A). The PHE group effectively restored the intestinal folds. Whole-mount Alcian blue staining revealed that PHE effectively counteracted the DSS-induced reduction in goblet cells, which were crucial for maintaining the physical barrier of the intestine (Figure 2C,D). In addition, qPCR analysis (Figure 2D) confirmed that PHE modulates the expression of the mucin gene *muc2.1*, a key component of the chemical barrier, thereby suggesting a regulatory mechanism at the genetic level. Moreover, PHE restored normal levels of D-lactic acid, indicating a reduction in DSS-induced intestinal permeability. Collectively, these findings (Figure 3F) underscored PHE’s capacity to mitigate DSS-treated intestinal damage by alleviating intestinal fold injury, reducing permeability, preserving goblet cell numbers, and maintaining MUC2 levels.

### 3.3. PHE Was Associated with Gut Microbiome Alterations

We further investigated the impact of PHE on the microbiome in our zebrafish DSS-induced intestinal inflammation model, revealing significant microbiota modulation associated with PHE treatment. The control group harbored 882 operational taxonomic units (OTUs), while the DSS and PHE groups had 599 and 537 OTUs, respectively, suggesting alterations in microbial richness among the groups. Figure 3A presents a Venn diagram that highlights the distinct microbial communities across the three experimental groups, with the majority of microorganisms being unique to each group. The alpha diversity indices, including the Chao (Figure 3B) and Simpson (Figure 3C) indices, showed no significant differences among the groups. Beta diversity analysis via Principal Coordinate Analysis (PCoA) (Figure 3D) further revealed distinct clustering of the PHE group, suggesting differences in microbial community structure. Community analysis at the class level, visualized through the Circos graph (Figure 3E), and variance analysis at the genus level (Figure 3F) revealed specific alterations in taxa associated with PHE treatment, including increases in potentially beneficial microbial populations. Notably, at the class level, Gammaproteobacteria were significantly reduced in the PHE group. This class includes many important pathogens, such as the genus Salmonella, which causes gastroenteritis and typhoid fever, suggesting a potential association with the alleviation of intestinal inflammation by PHE [11]. Additionally, Alphaproteobacteria showed an increase in the PHE group. The genus Pseudomonas was decreased in the PHE group, while the genera Microbacterium and Flectobacillus were elevated in the DSS group. Genera Allorhizobium, Neorhizobium, Pararhizobium, Rhizobium, and Phyllobacterium showed increases in the PHE group. LEfSe analysis (Figure 3G) identified key microbial biomarkers enriched in the PHE group, underscoring its role in microbiota reprogramming. Differences between groups showed that the control group contained families such as Micrococcales, Pseudomonadaceae, Halomonadaceae, Sphingomonadaceae, Nitriliruptoraceae, and Dietziaceae; the DSS group contained families such as Chitinophagaceae, Spirosomaceae, Rhizobiaceae, and Comamonadaceae; the PHE group contained families such as Microbacteriaceae and Rhizobiaceae. In the PHE group, the Rhizobiaceae family included genera Phyllobacterium and Pseudochrobactrum.

BugBase analysis (Figure 3H) indicated a reduction in potentially pathogenic bacteria in the PHE group compared to the DSS group. KEGG and functional predictions by PICRUSt2 (Figure 3I,J) suggested that PHE administration was associated with several predicted metabolic pathways, including those related to ATP-binding protein, iron complex transport system, peptide/nickel transport system permease protein, and amino acid transport and metabolism. These predictions are exploratory and require direct experimental validation. Collectively, these results demonstrate that PHE treatment is associated with beneficial modulation of the gut microbiome, which may contribute to the attenuation of DSS-induced intestinal inflammation in our zebrafish model, offering a promising avenue for further investigation.

### 3.4. PHE Displayed Potent Protective Effects Against DSS-Induced Oxidative Stress

**Figure 4:**
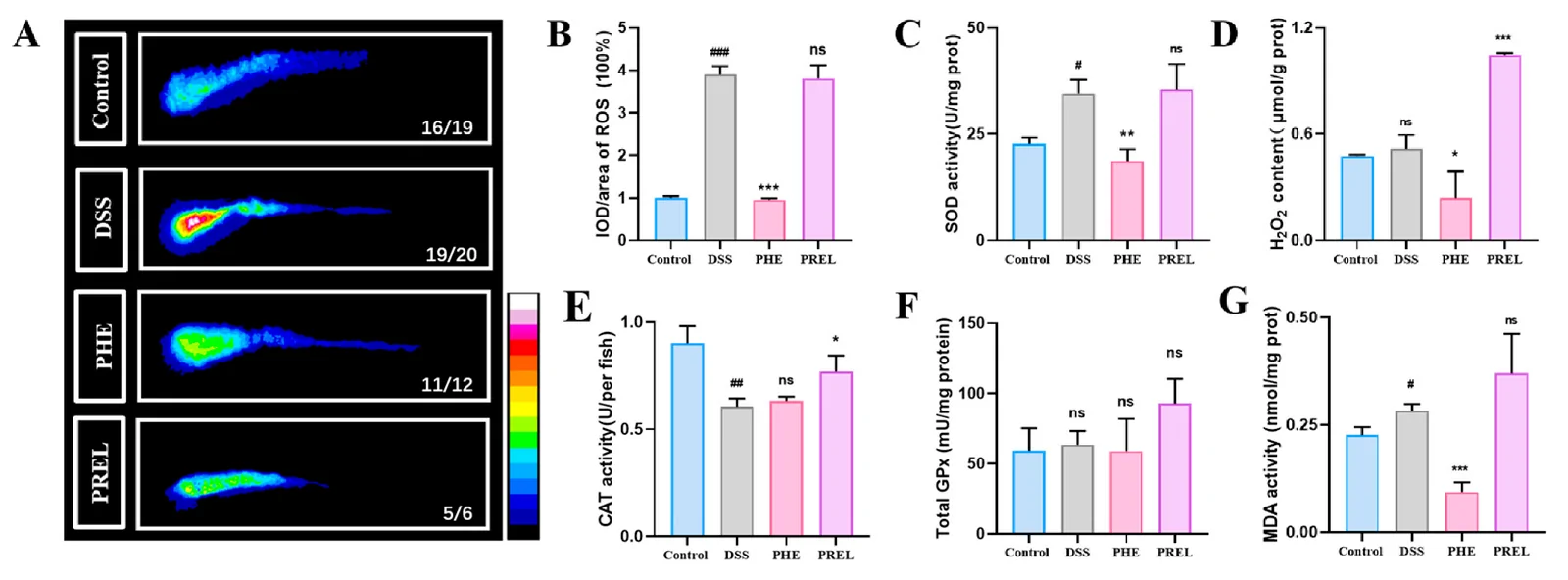
PHE supplementation rescued the impact of DSS on oxidative stress. (**A**) Coherency heatmaps (LUT 16-colors) showing ROS levels in different groups stained with DCFH-DA. (**B**) Quantification of fluorescence intensity from the ROS probe. (**C**–**G**) Measurement of SOD, H_2_O_2_, CAT, GPx, and MDA to assess oxidative stress levels. Data are presented as mean ± SD (n = 3 biological replicates per group). Multiple group comparisons were performed using one-way ANOVA followed by Tukey’s post hoc test. ### *p*  <  0.001, ## *p*  <  0.01, # *p*  <  0.05 versus the control group; *** *p*  <  0.001, ** *p*  <  0.01, * *p*  <  0.05 versus the DSS group; ns means no significance.

As illustrated in Figure 4A, coherency heatmaps generated through DCFH-DA staining revealed a significant reduction in ROS levels in the PHE-treated groups compared to the DSS group. Quantitative analysis of fluorescence intensity (Figure 4B) confirmed these findings, showing a substantial decrease in ROS levels after PHE administration.

Further assessment of five oxidative stress biomarkers across four groups (Figure 4C–G) was conducted. In the DSS group, SOD activity was notably increased, while CAT activity was significantly decreased and MDA levels were significantly elevated compared to the control group. Compared with the DSS group, the PHE-treated group exhibited significantly decreased SOD activity, as well as reduced H_2_O_2_ and MDA levels. The elevated SOD activity in the DSS group likely reflects a compensatory antioxidant response to increased ROS production. In the PHE-treated group, SOD activity decreased to levels comparable to the control group, suggesting restoration of normal antioxidant status rather than diminished scavenging capacity. The concurrent reduction in H_2_O_2_ levels further supports that the decreased SOD activity is associated with reduced oxidative burden, rather than direct inhibition of SOD by PHE. CAT activity did not show significant changes in the PHE group compared to the DSS group. Notably, MDA levels, a marker of lipid peroxidation, were significantly lower in the PHE-treated groups, further substantiating the protective role of PHE against oxidative damage. Collectively, these results emphasize the efficacy of PHE in mitigating DSS-induced oxidative stress, highlighting its potential therapeutic application in oxidative stress-related conditions.

### 3.5. Pharmacological Targets and Pathways for PHE Against IBD

**Figure 5:**
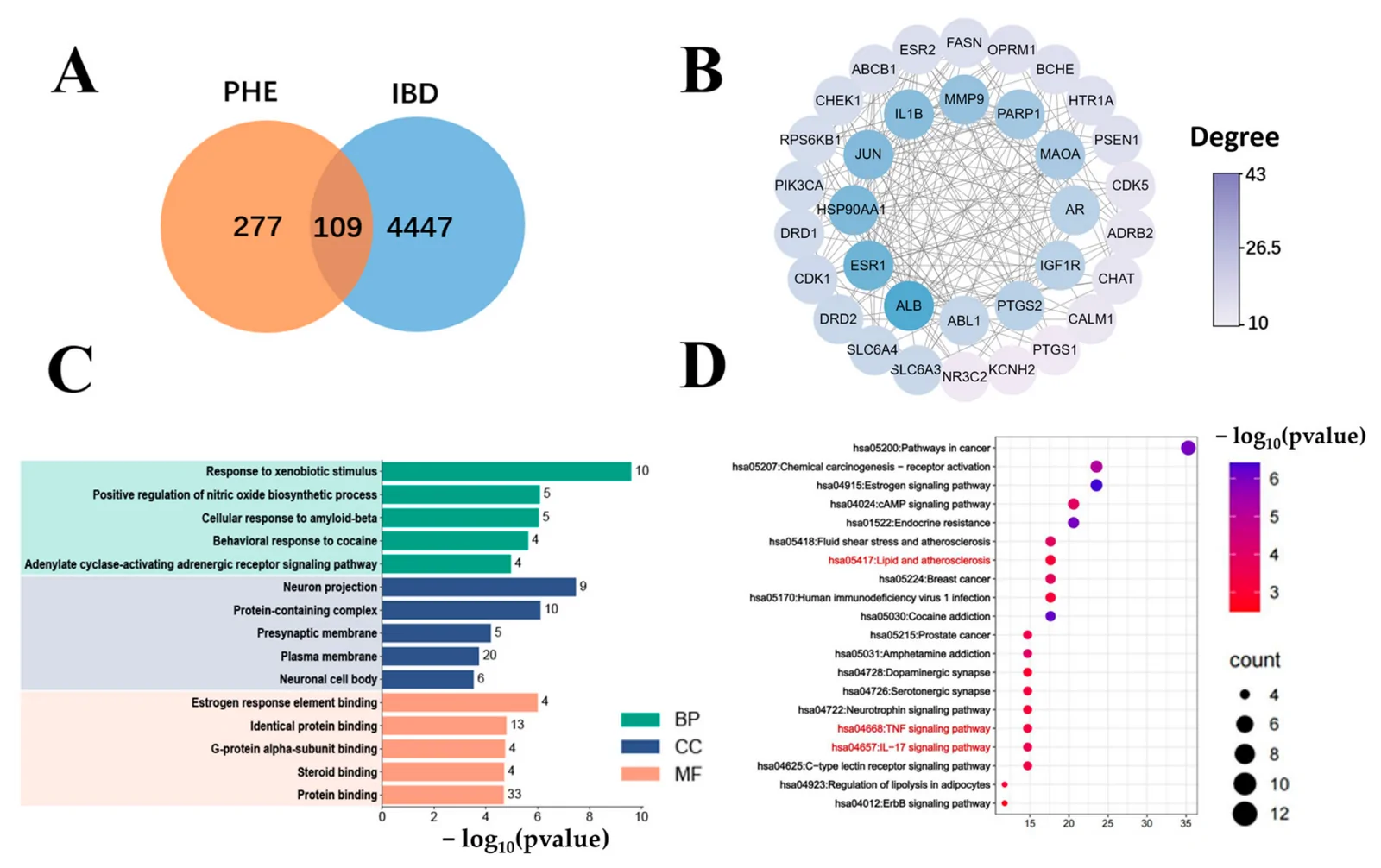
PHE target prediction for the treatment of IBD. (**A**) Illustration of a Venn diagram for PHE and IBD-related targets. (**B**) PPI network graph of core targets. The node color is proportional to its degree value. (**C**) Top 5 GO terms. (**D**) Bubble diagram of the top 20 KEGG enrichment results.

The collection and analysis of publicly available data identified a total of 386 potential targets associated with PHE and 4556 targets associated with IBD. (Figure 5A and Supplementary Material Tables S2 and S3). A Venn diagram (Figure 5A) depicted 109 common potential targets (Supplementary Material Table S4). Core targets (Figure 5B) were determined based on their exceedance of the median values for centrality, betweenness centrality, subgraph centrality, and closeness centrality (Supplementary Material Table S5).

A Gene Ontology (GO) enrichment analysis was performed with a significance threshold of *p* < 0.05, and the findings are detailed in Table S6. Figure 5C illustrates the leading five pathways linked to biological processes (BP), cellular components (CC), and molecular functions (MF). A subsequent Kyoto Encyclopedia of Genes and Genomes (KEGG) enrichment analysis identified 53 pathways with notable differences (*p* < 0.05), as recorded in Table S7. The top 20 pathways, ranked by *p*-value, are shown in Figure 5D. These pathways include the Estrogen signaling pathway, Cocaine addiction, Pathways in cancer, Endocrine resistance, Chemical carcinogenesis—receptor activation, Amphetamine addiction, cAMP signaling pathway, Fluid shear stress and atherosclerosis, Breast cancer, IL-17 signaling pathway, Prostate cancer, C-type lectin receptor signaling pathway, Serotonergic synapse, TNF signaling pathway, Neurotrophin signaling pathway, Human immunodeficiency virus 1 infection, Lipid and atherosclerosis, Dopaminergic synapse, Regulation of lipolysis in adipocytes, and the ErbB signaling pathway. The IL-17 signaling pathway, TNF signaling pathway, and Lipid and atherosclerosis were notably associated with IBD, warranting further validation through in vivo models.

### 3.6. Verification of the Three Potential Pathways Involved in PHE Alleviating DSS

**Figure 6:**
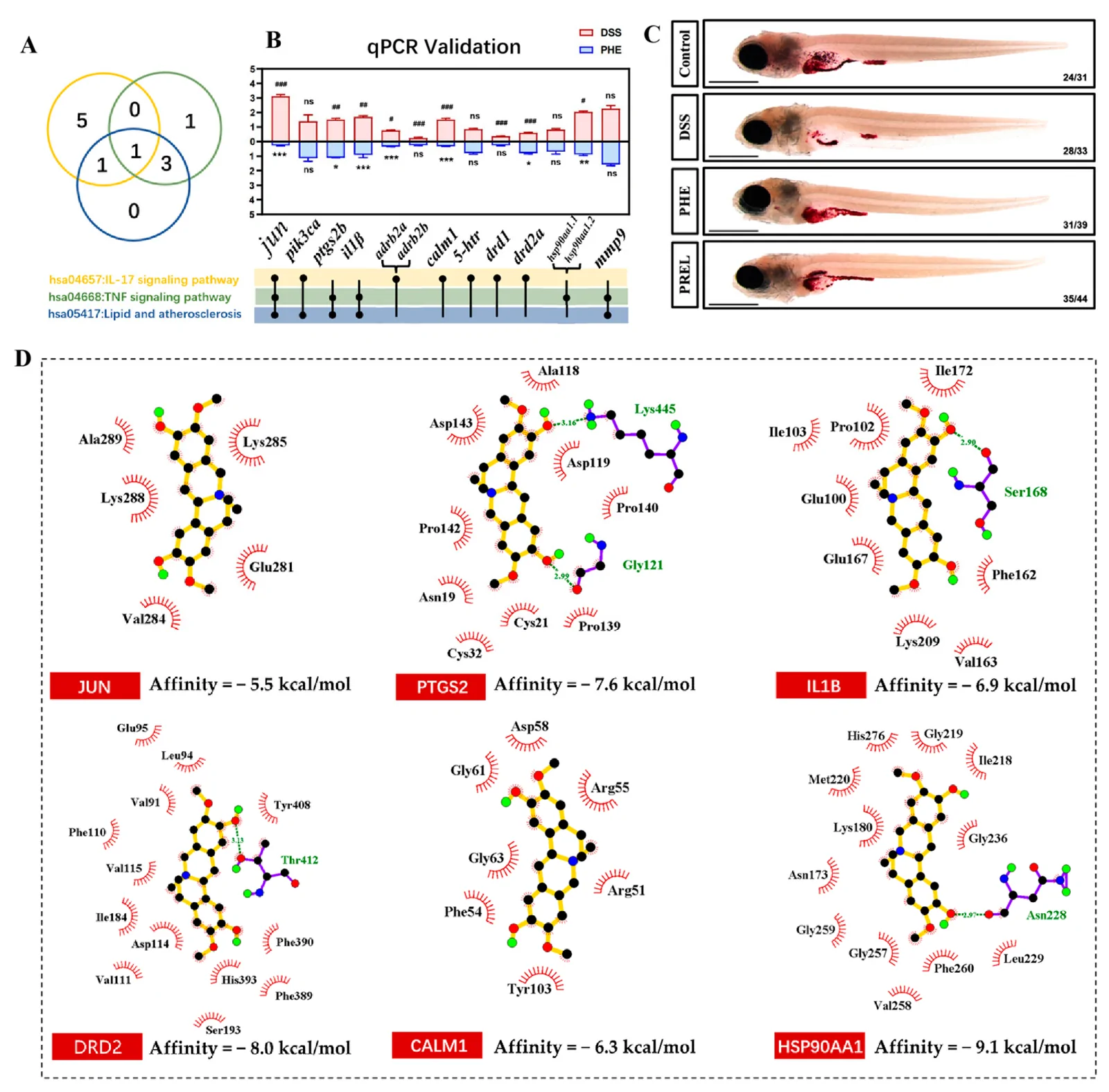
Target validations in vivo and docking prediction. (**A**) Venn diagram of the IL-17 signaling pathway, lipid and atherosclerosis, and TNF signaling pathway. (**B**) The mRNA expression levels of related genes in the three pathways. (**C**) Microphotograph of the ORO staining showing the accumulation of lipids in the intestines. (**D**) Structural simulation of PHE incorporation into the active site of the potential targets. Data are presented as mean ± SD (n = 3 biological replicates per group). Multiple group comparisons were performed using one-way ANOVA followed by Tukey’s post hoc test. (Molecular docking scores, kcal/mol). (### *p*  <  0.001, ## *p*  <  0.01, # *p*  <  0.05 versus the control group; *** *p*  <  0.001, ** *p*  <  0.01, * *p*  <  0.05 versus the DSS group; ns means no significance).

Eleven targets in the IL-17 signaling pathway, Lipid and atherosclerosis, and TNF signaling pathway were analyzed using a Venn diagram (Figure 6A). Jun proto-oncogene (JUN) emerged as the sole target present across all three pathways. The IL-17 signaling pathway contained five distinct genes, whereas the other two pathways had three shared targets. These eleven targets were subsequently confirmed via qPCR. Of these pathways, *jun*, *ptgs2*, *il1b*, *calm1*, and *hsp90aa1.2* were significantly upregulated in the DSS-induced model and recovered to normal or below normal levels in the PHE rescue group (Figure 6B). *adrb2a and adrb2b (zebrafish orthologs of mammalian ADRB2)*, *drd1*, and *drd2a* were down-regulated in the DSS-induced model. *adrb2a* was further down-regulated in the PHE rescue group; expression of *adrb2b* and *drd1* was not affected by PHE, and *drd2a* was upregulated by PHE. Furthermore, ORO staining (Figure 6C) revealed that intestinal lipid accumulation was markedly reduced in the DSS-induced group compared to the control group, indicating impaired lipid absorption capacity in the inflamed gut. Notably, both PHE and PREL treatment significantly restored lipid accumulation to levels approaching those of the control group, suggesting that PHE and PREL effectively recovered intestinal lipid absorption function. This recovery likely reflects the restoration of intestinal barrier integrity and overall gut metabolic function, rather than pathological lipid deposition. Molecular docking simulations (Figure 6D) provided structural insights into the incorporation of PHE into the active sites of potential targets, with docking scores supporting its binding affinity. Prostaglandin-endoperoxide synthase 2 (PTGS2), interleukin-1 beta (IL1B), dopamine receptor D2 (DRD2), and heat shock protein 90 alpha family class A member 1 (HSP90AA1) were predicted to have strong binding affinities for PHE, owing to forming hydrogen bonds (Figure 6D). These findings collectively highlight the therapeutic potential of PHE in modulating key pathways involved in inflammation and lipid metabolism, offering a promising avenue for future research in the treatment of related pathologies.

## 4. Discussion

This study provides a comprehensive characterization of the preventive effects of PHE in a zebrafish model of intestinal inflammation, encompassing intestinal barrier integrity, microbiome modulation, oxidative stress, and lipid absorption, while also employing network pharmacology to generate testable hypotheses for future research.

The DSS-induced IBD model is a well-established experimental setup that mimics several symptoms of human IBD, including immune dysregulation and disruption of the intestinal barrier [28]. Using a zebrafish model of DSS-induced intestinal inflammation, this study provides evidence that PHE exerts preventive effects through multiple mechanisms, suggesting its potential as a candidate for further investigation.

We observed that PHE ameliorated IBD symptoms, in part due to its anti-inflammatory property. PHE effectively reduced inflammatory cell infiltration in the intestinal tissue, which is a hallmark of IBD. PHE treatment resulted in M2-like macrophage polarization in vivo. M1-like macrophages are known for producing pro-inflammatory cytokines that exacerbate inflammation, whereas M2-like macrophages aid in tissue repair and the resolution of inflammation [1]. Results from our qPCR analysis of gene expression demonstrated that PHE downregulated the expression of key pro-inflammatory cytokines such as *ikbkb*, *NF-kB p65*, and *il1β*, while upregulating anti-inflammatory cytokines like *tgfb1a*. This modulation of cytokines indicates that PHE not only inhibited the onset of inflammation but also promoted the resolution phase, positioning it as a promising candidate for the treatment of IBD.

Furthermore, the DSS-treated group exhibited reduced mucin-producing goblet cells and abnormalities in intestinal permeability compared to the healthy group. The intestinal mucus barrier, secreted by goblet cells and enveloping the epithelial cell layer, serves as a defense against bacterial invasion and is crucial for preserving intestinal homeostasis [29,30]. Notably, PHE restored the integrity of the intestinal barrier, as evidenced by the preservation of goblet cells and *muc2.1* expression, underscoring its importance in maintaining mucosal homeostasis. Disruption of chemical and physical barriers is closely related to immune infiltration [31]. The repairs in both barriers mediated by PHE likely account for the resolution of inflammation. The main constituent of the mucus layer is MUC2, a highly O-glycosylated molecule that assembles into polymeric sheets [32]. These sheets act as attachment sites to capture microorganisms, inhibiting their colonization of the intestinal epithelium, while also providing a nutrient source for the gut microbiota [32]. Consequently, augmentation of the mucus barrier mediated by PHE may improve the intestinal environment for the microbiota. We acknowledge that our barrier assessment was primarily based on MUC2 staining, goblet cell counts, and permeability assays. While the current data consistently support the protective role of PHE in barrier integrity, we recognize that additional markers—such as tight junction proteins and histological scoring—would further strengthen this conclusion and are worth exploring in future studies. In this study, we considered goblet cells as components of the physical epithelial layer and MUC2 as the main structural component of the chemical mucus barrier, reflecting their cellular and secreted nature, respectively.

To gain insight into the changes in microbial composition, the microbiome profiles of the healthy, DSS, and PHE groups were compared. Our metagenome analysis revealed that PHE treatment was associated with a significant modulation of the microbiota. These alterations were characterized by a reduction in potentially pathogenic bacteria and an increase in beneficial microbial populations. Notably, at the class level, there was a significant decrease in Gammaproteobacteria within the PHE group. This class encompasses numerous critical pathogens responsible for gastroenteritis and typhoid fever, suggesting a potential association with the alleviation of intestinal inflammation. Furthermore, Flectobacillus, a genus of Gram-negative bacteria, had a positive correlation with high-fat diet-induced intestinal inflammation in zebrafish, and this genus exhibited a significant increase in the DSS group [33]. However, it showed a reduction in the PHE group, suggesting a potential association with the improvement of intestinal inflammation. Given the recognized role of the microbiota in IBD pathogenesis, the microbial alterations associated with PHE treatment may contribute to its protective effects, offering valuable insights for future mechanistic studies. We acknowledge that negative extraction controls, water controls, and reagent controls were not included in our microbiome analysis. However, all larvae were thoroughly washed with sterile E3 medium and processed in parallel to minimize contamination, ensuring reliable comparisons across groups. Future studies incorporating these controls would provide further validation.

PICRUSt2 predictions suggested that PHE administration was associated with several metabolic pathways, including those related to ATP-binding proteins, iron complex transport systems, peptide/nickel transport system permease proteins, and amino acid transport and metabolism, which may be associated with the resolution of intestinal inflammation. These predicted functional changes warrant further experimental validation. In inflamed intestinal areas, activated macrophages and neutrophils produce excessive ROS [34]. We observed the activation of oxidative stress in the intestine in response to a 3-day DSS treatment. PHE significantly attenuated markers of oxidative stress, such as ROS and H_2_O_2_. This is further supported by the observed decrease in the MDA level, a marker of lipid peroxidation, indicating reduced oxidative damage to cellular membranes [35]. These observations suggest that the preventive effects of PHE against intestinal inflammation may be partly attributed to the attenuation of excessive oxidative stress, which is known to play a critical role in intestinal inflammation by promoting tissue damage and sustaining inflammatory responses [34]. Considering that oxidative stress is recognized as being able to disrupt tight junctions and enhance intestinal permeability, the antioxidant properties of PHE may also contribute to its ability to preserve the integrity of the intestinal barrier [36].

DSS-induced colitis has been reported to disrupt multiple metabolic processes including fatty acid oxidation, lipogenesis, and bile acid synthesis, while simultaneously impairing intestinal barrier function [37]. Given that an intact intestinal barrier is a prerequisite for efficient nutrient absorption, the reduced lipid accumulation observed in our DSS group likely reflects impaired lipid absorption secondary to barrier disruption. Notably, natural products such as Atractylodes macrocephala polysaccharide have been shown to alleviate DSS-induced colitis through coordinated regulation of lipid metabolism and intestinal barrier integrity [38], which parallels our observations with PHE treatment.

To investigate the specific mechanisms of the anti-IBD effects of PHE, we combined network pharmacology and in vivo validation methods. Our analysis identified 109 common targets between PHE and IBD. Following the GO and KEGG analyses, along with qPCR validation, we concentrated on six pivotal targets: JUN, PTGS2, IL1B, DRD2, calmodulin 1 (CALM1), and HSP90AA1. The role of JUN proteins, a component of the AP-1 transcription factor, is involved in regulating inflammatory responses and cell proliferation [39]. Therefore, the influence of PHE on JUN indicates its involvement in the regulation of pro-inflammatory cytokine expression and the facilitation of tissue repair. Previous studies have shown that PTGS2, also known as COX-2, is a well-known mediator of inflammation and pain, and its downregulation by PHE aligned with the observed reduction in inflammatory markers [40,41]. IL1B is a pro-inflammatory cytokine, which is directly involved in the inflammatory cascade, and its regulation by PHE further supports its anti-inflammatory effects [42]. DRD2 modulates the immunosuppressive function and colonic homing of regulatory T (Treg) cells. Activation of DRD2 enhances Treg suppressive activity and promotes their recruitment to the inflamed colon, whereas DRD2 deficiency in T cells is associated with exacerbated colitis [43]. Notably, DRD2A, a subtype of DRD2, was significantly modulated by PHE in our experimental model, suggesting that the protective effects observed may involve this DRD2-mediated immunoregulatory axis. CALM1, a calcium-binding protein, plays a role in various cellular processes, including inflammation and apoptosis, and its regulation by PHE may contribute to maintaining cellular homeostasis [44]. HSP90AA1, a molecular chaperone, is involved in protein folding and stress responses, and its modulation by PHE may enhance cellular resilience to stress [45]. These results suggest the involvement of multiple pathways in the effects of PHE observed in our zebrafish model, providing a useful basis for future investigations. By identifying these candidate targets, our study offers valuable clues for further exploration of the molecular basis of PHE’s action and its potential as a multi-target agent, although these preliminary findings require further experimental validation.

**Figure 7:**
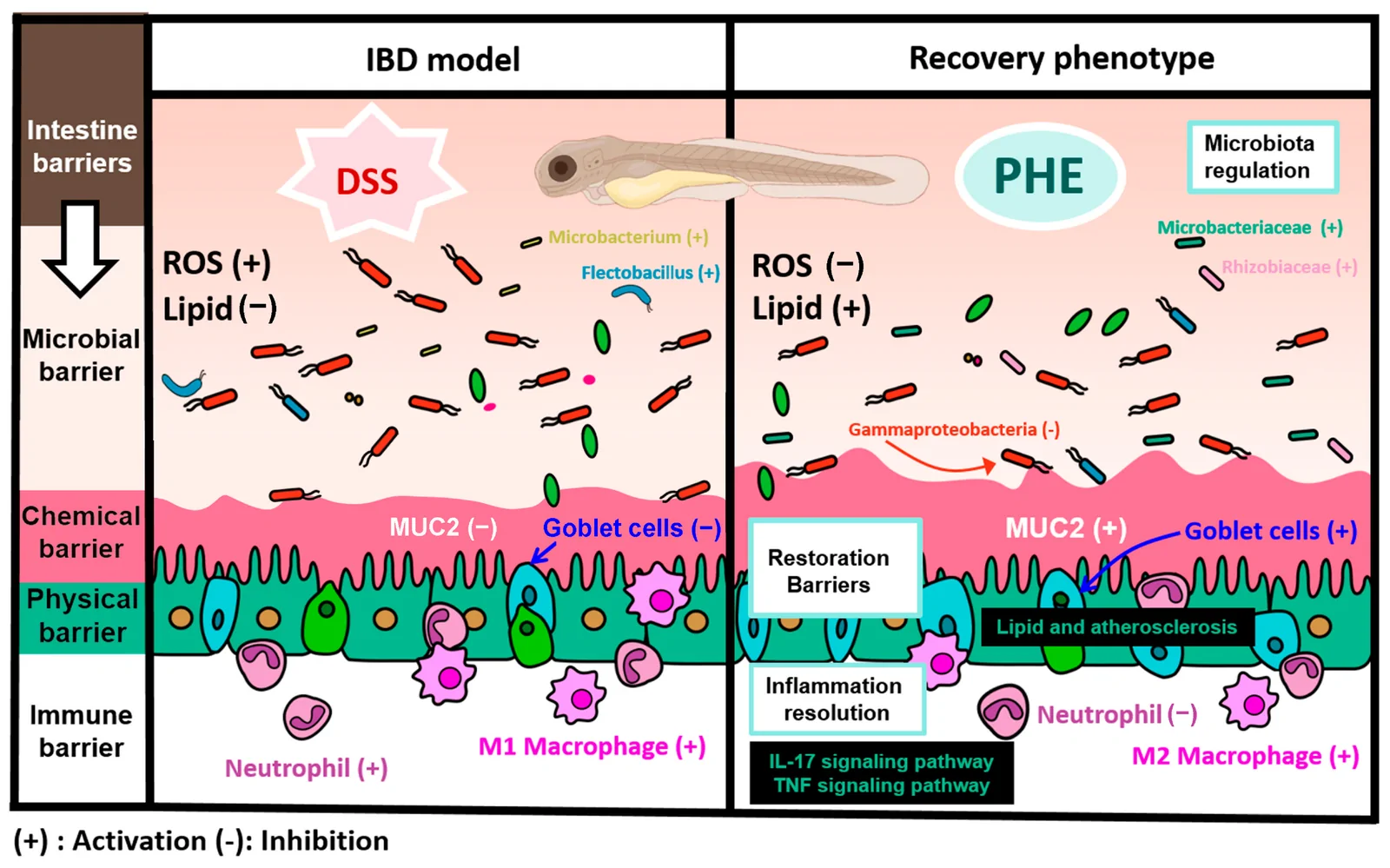
The schematic diagram illustrates key mechanisms involved in PHE alleviation of IBD via PHE-mediated repair of intestinal barriers (microbial, chemical, physical, and immune barriers): Reducing ROS production and promoting lipid accumulation promotes regulation of the microbiota. Counteracting DSS-induced reduction in Goblet cells restores the chemical barrier. Resolution of inflammation restores the physical barrier and the immune barrier.

We acknowledge several limitations in this study. The zebrafish larval model, while advantageous for initial screening, may not fully reflect human IBD pathology, and our findings warrant validation in mammalian models. The network pharmacology and molecular docking results are hypothesis-generating and require experimental confirmation. Additionally, our gene expression data are limited to the transcript level, and protein-level verification would be a valuable next step. Despite these limitations, we believe our multifaceted approach provides a reliable preliminary assessment of PHE’s protective potential in intestinal inflammation. In summary, our findings in the zebrafish model suggest that PHE attenuates DSS-induced intestinal inflammation through multiple mechanisms, including resolution of inflammation, reduction in ROS production, and restoration of intestinal lipid absorption capacity (Figure 7). These effects were associated with improvements in the four intestinal barriers (microbial, chemical, physical, and immune). Network pharmacology and gene expression analyses further pointed to the IL-17 signaling pathway, the lipid and atherosclerosis pathway, and the TNF signaling pathway as potential mediators of the observed protective effects. Collectively, these results provide preliminary evidence for the preventive effects of PHE in the zebrafish model and offer a foundation for future investigations into its mechanisms of action.

## 5. Conclusions

This study suggests that PHE exerts preventive effects against DSS-induced intestinal inflammation in a zebrafish model through multiple mechanisms. PHE demonstrated its effectiveness in reducing immune cell infiltration and alternatively polarized macrophages. Additionally, it restored intestinal barriers, thereby enhancing gut integrity. PHE also reshaped microbiome profiles, contributing to a healthier microbial environment. Furthermore, it mitigated oxidative stress, which is critical in protecting intestinal tissues from damage. The identification of key molecular targets, including JUN, PTGS2, IL1B, CALM1, DRD2, and HSP90AA1, provided valuable insights into the mechanisms underlying PHE’s action. These findings suggest that PHE exerts preventive effects against DSS-induced intestinal inflammation in a zebrafish model, potentially through multiple protective mechanisms.

## Data Availability

The data presented in this study are available on request from the corresponding author.

## Supplementary Materials

The following supporting information can be downloaded at: https://www.mdpi.com/article/10.3390/biom16081092/s1, Text S1: Docking preparation; Figure S1: RT-qPCR analysis of potential targets; Table S1: Primer sequences used in the study; Table S2: Predicted targets of PHE; Table S3: IBD targets; Table S4: 109 common targets; Table S5: 34 key targets; Table S6: GO analysis; Table S7: KEGG analysis.
