# Supplementary material for: Unraveling Effects and Pharmacological Mechanisms of Phellodendrine on Inflammatory Bowel Disease
Source: Biomolecules. 2026 Jul 26;16(8):1092. doi: 10.3390/biom16081092 (PMC13510209; doi:10.3390/biom16081092)
Supplement: Supplementary file 1 [file biomolecules-16-01092-s001.zip › Supporting Information-PHE.pdf]

Supplementary data

Unraveling Effects and Pharmacological Mechanisms of Phellodendrine on Inflammatory Bowel Disease

Yufeng Xie [1,†], Ziyi Zhou [1,†], Xuqianzi Wu [1], Jiayin Teng [1], Xiaorun Zhang [1], Yue Sun [1], Lixin Chen [1], Lijian Ding [2,*] and Wei Yuan [1,*]

[1] Jiangxi Provincial Key Laboratory of Synthetic Pharmaceutical Chemistry, School of Geography and Environmental Engineering, Gannan Normal University, Ganzhou 341000, China; 1241144005@gnnu.edu.cn (Y.X.)
[2] School of Pharmacy, Health Science Center, Ningbo University, Ningbo 315211, China
* Correspondence: dinglijian@nbu.edu.cn (L.D.); 1400046@gnnu.edu.cn (W.Y.)
[†] These authors contributed equally to this work.

Text S1: Docking preparation

The three-dimensional conformations of six target proteins were retrieved from the PDB protein database (http://www.pdb.org/). The docking process involved the following proteins: Jun proto-oncogene (JUN, PDB: P05627.1.A), prostaglandin-endoperoxide synthase 2 (PTGS2, PDB: P35354.1.A), interleukin 1 beta (IL1B, PDB: P46648.1.A), calmodulin 1 (CALM1, PDB: P0DP24.1.A), dopamine receptor D2 (DRD2, PDB: P14416.1.A), heat shock protein 90 alpha family class A member 1 (HSP90AA1, PDB: A0A2I3S281.1.A). The PyMOL software version 1.7.1.0 was utilized to eliminate heteroatoms and water molecules from the protein structures. The PyMOL software version 1.7.1.0 was utilized to eliminate heteroatoms and water molecules from the protein structures.

Figure S1: RT-qPCR analysis of potential targets.

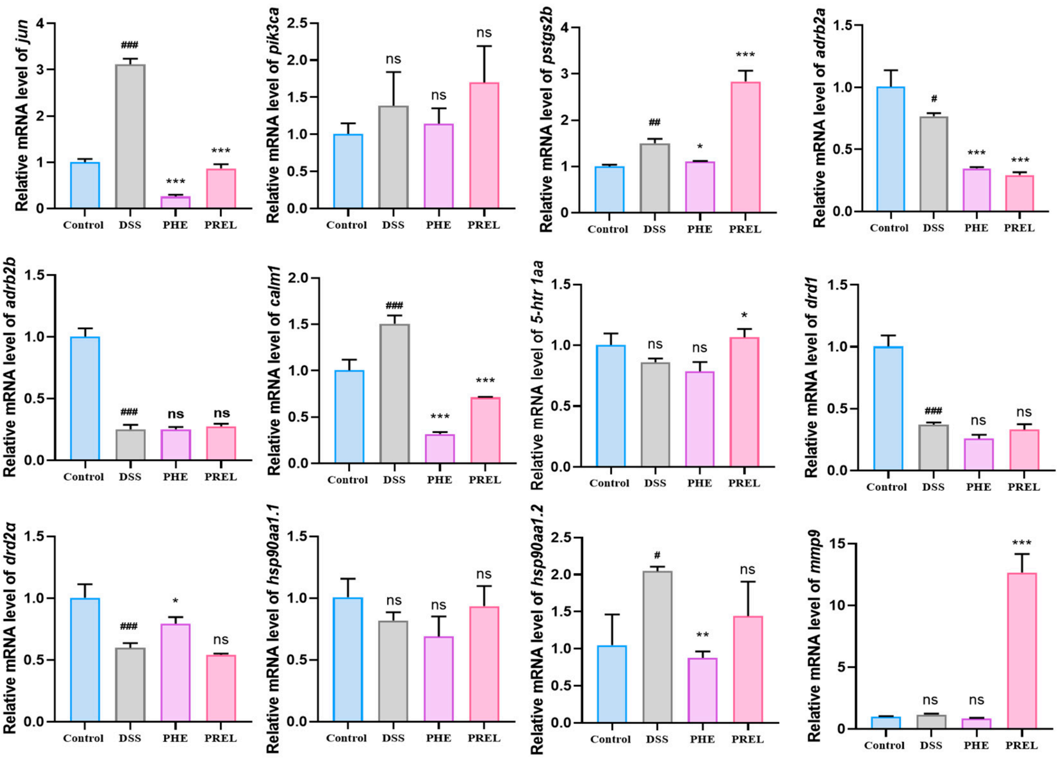

Figure S1: qPCR validations of potential targets in key pathways (###p < 0.001, ##p < 0.01, #p < 0.05 versus the control group; ***p < 0.001, **p < 0.01, *p < 0.05 versus the DSS group; ns means no significance. Mean ± SD).
