# Supplementary material for: Unraveling Effects and Pharmacological Mechanisms of Phellodendrine on Inflammatory Bowel Disease
Source: Biomolecules. 2026 Jul 26;16(8):1092. doi: 10.3390/biom16081092 (PMC13510209; doi:10.3390/biom16081092)
Supplement: Supplementary file 1 [file biomolecules-16-01092-s001.zip › Table S1 Primer sequences used in the study.pdf]

Table S1. Primer sequences used in the study

Gene name	Sequences of the primer (5' - 3')	Sequences of the primer (5' - 3')
ikbkb	F:ACCAGCATTCAGATCGCCATC	R:TCTCGCCGCAACCACATTCAT
NF-kB p65	F:CAGAGTGCGGATCTCCTTGG	R:ATGGGTTGTTTTGCGTCTGC
il1β	F:GGACTGTTCAGATCCGCTTGC	R:TCCACCACGTTCACTTCACG
tgfβ1a	F:AGACCTGCTGTATGCGCAAGCTTTAC	R:ACCATGTTGGACAATTGCTCCACCTTG
muc2.1	F:CCAAGATGAAGGACAAGGAGAG	R:GGAGAAAACCGTACACAGACA
jun	F:ACGTGGGACTTCTCAAACTG	R:TCTTGGGACACAGAAACTGG
pik3ca	F:CGCAATGAGAGGATGAGCGA	R:ACGCTGTCACGATGGAACAA
pstgs2b	F:GGCTCATCCTTATTGGTGAGACTAT	R: TCGGGATCAAACTTGAGCTTAAAATA
adrb2a	F:GTCACGCTATCCTAACGTCA	R:ATTCCTCTTTCGCCAAGTTC
adrb2b	F:AAGCCTTTGAACCAAGATG	R:GCCTTTCCAAATATGTCCTG
calm1	F:TACATGCATGCAGCTTGAGT	R:ATTGAAGCAAACTGCAAAGG
5-htr 1aa	F:GTCATCGCTGCTATCGCCTTGG	R:GCACCAGCACCGACACCATAAG
drd1	F:ACGCTGTCCATCCTTATCTC	R:TGTCCGATTAAGGCTGGAG
drd2α	F:TGGTACTCCGGAAAAGACG	R:ATCGGGATGGGTGCATTTC
hsp90aa1.1	F:CAATGATGACGAGCAG	R:AGGGTAGCCAATGAAC
hsp90aa1.2	F:GCTGAACAAAACCAAACC	R:AAAGAGTAGGGCACGGA
mmp9	F:GCTCAACCACCGCAGACTAT	R:GTGCTTCATTGCTGTTCCCG
ef1a	F:GCTCAAACATGGGCTGGTTC	R: AGGGCATCAAGAAGAGTAGTACCG
